# Reprocessamento de Marca-Passos em Países de Língua Portuguesa: Uma Reflexão Clínica

**DOI:** 10.36660/abc.20210941

**Published:** 2023-03-01

**Authors:** Neiberg de Alcantara Lima, Eduardo Arrais Rocha, Albertino Damasceno, Ieda Prata Costa, José Ribeiro Bunda Ricardo, Fernando Jorge Lopes, Luis Dias, Miryan Bandeira dos Prazeres Cassandra Soares, Eric Puroll, Kim A. Eagle, Thomas C. Crawford

**Affiliations:** 1 Wayne State University School of Medicine Detroit Michigan EUA Wayne State University School of Medicine – Internal Medicine, Detroit, Michigan – EUA; 2 Universidade Federal do Ceará Hospital Universitário Walter Cantídio Faculdade de Medicina Fortaleza CE Brasil Universidade Federal do Ceará – Hospital Universitário Walter Cantídio – Programa de Pós-graduação em Ciências Cardiovasculares da Faculdade de Medicina da UFC, Fortaleza, CE – Brasil; 3 Universidade Eduardo Mondlane Faculdade de Medicine Maputo Moçambique Universidade Eduardo Mondlane – Faculdade de Medicine, Maputo – Moçambique; 4 Clinica Girassol Luanda Angola Clinica Girassol, Luanda – Angola; 5 Hospital Dr. Baptista de Sousa Mindelo Cabo Verde Hospital Dr. Baptista de Sousa, Mindelo – Cabo Verde; 6 Hospital Agostinho Neto Praia Cabo Verde Hospital Agostinho Neto, Praia – Cabo Verde; 7 Hospital Dr. Ayres de Menezes São Tomé São Tomé e Príncipe Hospital Dr. Ayres de Menezes, São Tomé – São Tomé e Príncipe; 8 University of Michigan Medicine Ann Arbor Michigan EUA University of Michigan Medicine, Ann Arbor, Michigan – EUA; 9 University of Michigan Medicine Frankel Cardiovascular Center Ann Arbor Michigan EUA University of Michigan Medicine – Frankel Cardiovascular Center, Ann Arbor, Michigan – EUA

**Keywords:** Estimulação Cardíaca Artificial/tendências, Marca-Passo Artificial, Desfibriladores Implantáveis, Administração em Saúde Pública, Disparidade nos Níveis de Saúde

## Abstract

Há uma enorme disparidade entre os países de alta renda e outros em termos de acesso a dispositivos médicos cardíacos, como marca-passos e desfibriladores implantáveis. Os custos são uma das principais barreiras ao uso de dispositivos cardíacos nesses países. Existem iniciativas internacionais que visam reduzir essa disparidade, e o reuso de marca-passos tem sido discutido como uma possível alternativa. O conceito de reutilização de marca-passos não é novo; entretanto, estudos recentes têm se mostrado seguros, éticos e eficazes para aqueles que precisam de dispositivos eletrônicos cardíacos implantáveis e não tem como adquiri-los. Parte dos países de língua portuguesa, especialmente na África, precisam de uma resposta imediata que beneficie seus inúmeros pacientes que sofrem de arritmias tratáveis.

## Introdução

A estimulação cardíaca artificial e a eletrofisiologia são atividades médicas estabelecidas e reconhecidas internacionalmente, porém ainda há um abismo no acesso aos diversos tratamentos entre países de alta renda e os demais. A reutilização de marca-passos vem sendo discutida como uma possível alternativa a essa problemática.^1^ Contudo, mesmo havendo uma necessidade evidente, países de baixa e média renda da língua portuguesa ainda não possuem políticas públicas ou começaram discussões amplas em suas sociedades médicas sobre essa temática. Este artigo se propõe a revisar e resumir o que se conhece sobre essa temática, particularmente em regiões de maior dificuldade de acesso a esses aparelhos.

### Disparidades em estimulação cardíaca artificial

Desde os anos 1950, quando os primeiros marca-passos foram implantados, até agora, houve um avanço sem precedentes no tratamento das doenças cardíacas.^2^ Apesar de a tecnologia ter avançado bastante, ela ainda é cara e inacessível para muitos países de baixa e média renda. Para se ter um exemplo, a taxa de implante anual de marca-passo é de mais de 700 por milhão de habitantes na França, Suécia e Estados Unidos, enquanto chega a ser menos de 7 por milhão no Paquistão, Filipinas e Indonésia e menos de 3 por milhão na África. Baseado nesses números, é estimado que até um milhão de pessoas morram todos os anos sem acesso a terapia para bradicardia no mundo.^3 - 5^ Os custos são uma das principais barreiras para à ampla utilização de dispositivos cardíacos nesses países. Um marca-passo chega a custar 2500 dólares americanos, isso é várias vezes mais do que a renda per capita de muitos países de baixa e média renda.^5^A falta de políticas públicas para doenças crônicas não infecciosas, profissionais capacitados para reconhecer e tratar arritmias cardíacas, acesso da população carente a hospitais especializados e de infraestrutura cirúrgica é outra possível razão.

Existem iniciativas que visam diminuir esse abismo entre países de alta renda e países de baixa e média renda, como o *Heartbeat International,* uma organização que distribui marca-passos próximos ao vencimento, que são doados generosamente pela indústria de marca-passos.^6^

O Projeto “ *My Heart Your Heart”* (PMHYH) é uma colaboração entre cidadãos, médicos e diretores de funerárias dos Estados Unidos, a Faculdade de Medicina da Universidade de Michigan, a *NEScientific* e a *World Medical Relief (WMR)* . A WMR é uma organização filantrópica sem fins lucrativos, com sede em Detroit, cuja missão é afetar o bem-estar de indivíduos pobres em escala internacional por meio da distribuição de suprimentos médicos, equipamentos e remédios doados.^7^ O objetivo da colaboração é determinar se a reutilização do marca-passo pode se mostrar um meio seguro de prestar cuidados a pacientes em países de baixa e média renda.

Antes da aprovação de ensaios clínicos internacionais, o projeto MHYH passou os primeiros anos fornecendo a estrutura para que essa ideia fosse apoiada por todos aqueles que ela afetaria.

Em mais de 10 anos, o projeto já beneficiou inúmeros pacientes que receberam de forma gratuita marca-passos recondicionados em países como Filipinas, Venezuela, Quênia e Serra Leoa, entre outros. O programa também fornece treinamento e acompanhamento de profissionais locais e pacientes. Para mais informações, acesse *http://www.myheartyourheart.org.*

### Reutilização de dispositivos cardíacos

O conceito de reutilização de marca-passos não é novo.^1^ Apesar de nunca terem sido a norma padrão, eles já foram utilizados no Brasil e Índia na década de 80 e 90.^8 , 9^ Na década de 90, a Suécia reutilizava cerca de 5% dos seus aparelhos.^10^ Tentativas de doação de marca-passos retirados de doadores cadáveres para países em desenvolvimento por médicos americanos data da década de 80.^11^ Cardiodesfibriladores implantáveis (CDIs) e marca-passos biventriculares reutilizados também vêm sendo utilizados há algum tempo na África, porém em menor escala.^12 , 13^

As fontes potenciais de doação de marca-passos são: *post-mortem* e pós-extração. Um estudo mostrou que mais de três mil aparelhos são retirados por crematórios americanos, sendo que mais de um quinto tinha bateria restante superior a 4 anos e que um aparelho novo tem bateria com longevidade de até 12 a 14 anos, variando de acordo com a programação e o fabricante.^14^Mais de sessenta mil aparelhos são explantados por ano nos Estados Unidos, e há uma estimativa de que cerca de 20% destes poderiam ser reutilizados, gerando um total anual de 10 a 12 mil aparelhos-ano.^15 - 17^

Embora a doação de geradores de marca-passos seja viável, a reutilização de eletrodos é bem mais difícil. Eletrodos são, em geral, difíceis de serem retirados devido ao processo de fibrose causado ao longo do tempo após o seu implante, e a sua extração é potencialmente danosa a eles. Mesmo admitindo que em situações pontuais esses eletrodos possam ser adequadamente retirados, o processo de esterilização de eletrodos é muito mais desafiador.^18^ Entretanto, eletrodos de marca-passo são bem mais baratos que os geradores, podendo ser mais facilmente adquiridos por países de baixa e média renda e até mesmo por seus habitantes.

### Segurança

Quando se fala no uso de um aparelho reprocessado, as principais preocupações são o risco de infecção e o risco de disfunção dos aparelhos ( Tabela 1 ).

**Tabela 1:** – Segurança dos aparelhos reprocessados

ID do Estudo	Anos do Estudo	Tipo de estudo	País	Total de pacientes com aparelhos reprocessados	Taxa de Infecção (%)	Taxa de Disfunção de dispositivo (%)	Mortalidade
Panja M et al. Indian Heart J, 1996^* 9*^	1976-1992	Observacional	Índia	642	11,8% *A maioria quando os dispositivos eram reutilizados no mesmo paciente após serem esterilizados.*	3,1% * - Altos limiares *	Não reportada
Baman T et al. Circ Arrhythm Eletrophysiol 2011 ^*21*^	2011	Metanálise	Índia, Romênia, Suécia, Brasil, Hungria, Israel, Austrália, Finlândia, Noruega, Canadá, Holanda, Filipinas, Itália	2270	1,76%. * Sem diferença do grupo controle. *	0,5%. *Maior que no grupo de aparelhos novos. *	Nenhum
Enache et al. PACE, 2019 ^*22*^	2001-2012	Retrospectivo. Caso controle.	Romênia	157	5% *Sem diferença do grupo controle*	Nenhum	Nenhum
Sethi et al. Indian Heart J. 1992 ^*19*^	1979-1991	Observacional	Índia	42	2,3%	Nenhum	Nenhum
Khairy et al. NEJM, 2020 ^*23*^	2003-2017	Controlado. Prospectivo.	Cuba, República Dominicana, Equador, Guatemala, Honduras e México	1051	2% *Sem diferença do grupo controle*	Nenhum	Nenhum
Baman et al. JACC 2009 ^*7*^	2008	Série de Casos	Filipinas	12	Nenhum	Nenhum	Nenhum
Sinha et al. PACE 2018^*4*^	2009-2017	Metanálise	Filipinas, Nicaragua, Índia, México, China, África do Sul, Romênia, Índia.	856	2,1%. *Sem diferença do grupo controle*	0,2% *Sem diferença do grupo controle*	Nenhum

Em relação a infecção, há uma vasta literatura mostrando que aparelhos recondicionados são usados com segurança. Entre 1979 e 1991, quarenta e dois aparelhos foram doados e reutilizados na Índia após serem explantados, em sua maioria devido a infecção de gerador, e, após um seguimento de 3 anos, apenas 2 pacientes tiveram complicações infecciosas.^19^

Na década de 80, na cidade de São Paulo, 22 aparelhos foram reutilizados após descontaminação química com o uso de óxido etílico. Desses pacientes, um teve complicação eletromagnética devido à inibição do gerador que aconteceu 2 meses após o implante.^20^

Em 2011, uma metanálise que tinha a segurança do reuso de marca-passos como desfecho primário, incluiu 18 trabalhos publicados de 1988 a 2008, totalizando 2270 pacientes. Os métodos de reprocessamento não foram homogêneos. A taxa de infecção foi de apenas 1,97%. Desses 18 trabalhos, 5 foram controlados e totalizaram 913 pacientes, mostrando ausência de diferença de infecção, em comparação com 6697 novos implantes de aparelhos.^21^

Um estudo não randomizado sobre a reutilização de CDIs comparou 157 pacientes que receberam aparelhos reutilizados e 114 que receberam novos aparelhos. Não houve diferença estatística entre os grupos no que se refere a infecções, disfunção de aparelho ou esgotamento inesperado de bateria.^22^

Em 2020, um grupo da Universidade de Montreal com participação internacional publicou um importante estudo no *New England Journal of Medicin* e que comparou 1051 aparelhos de marca-passo e CDI implantados no México, República Dominicana, Guatemala e Honduras com 3131 implantes de marca-passos novos realizados no Canadá. As características basais dos pacientes controles no Canadá foram ajustadas para o grupo do estudo. Os protocolos de esterilização dos marca-passos não foram descritos. Não houve diferença estatística de mortalidade relacionada ao aparelho ou infecção entre os grupos.^23^

A mesma metanálise de 2011 teve como desfecho secundário a avaliação de disfunção de marca-passo e incluiu dezessete trabalhos, totalizando 2150 aparelhos reutilizados, mostrando apenas 13 disfunções de aparelho, descritos como “erros técnicos” (n=5), esgotamento prematuro de bateria (n=1), reprogramação espontânea (n=1), inibição eletromagnética (n=1), inibição por músculo peitoral (n=1), anormalidades nos parafusos (n=3) e taquicardia ventricular espontânea (n=1). Em quatro estudos controlados dessa metanálise de 793 marca-passos reutilizados e 2200 novos implantes, houve um aumento no risco de disfunção nos aparelhos reutilizados.^21^

A maior desvantagem da reutilização de dispositivos, naturalmente, é a vida útil inferior ao aparelho novo, que já é um evento esperado.^21^ O Projeto MHYH somente processa aparelhos se a longevidade exceder quatro anos ou 75% da bateria de fábrica.

### O reprocessamento

**Figura 1:**
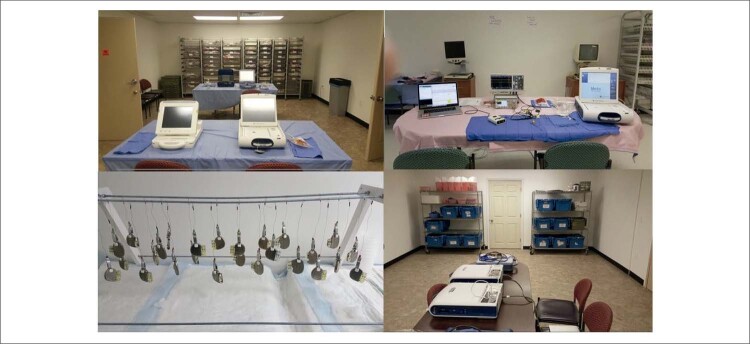
– Projeto My Heart your Heart in Detroit.

Para diminuir o risco de infecção e disfunção do aparelho já há técnicas validadas para o reprocessamento de aparelhos de marca-passo cardíaco. Aqui descrevemos a técnica desenvolvida pelo nosso grupo na Universidade de Michigan e publicada no *Journal of American College of Cardiology* em 2017 ( Figura 1 ).^24^

**Figura 2:**
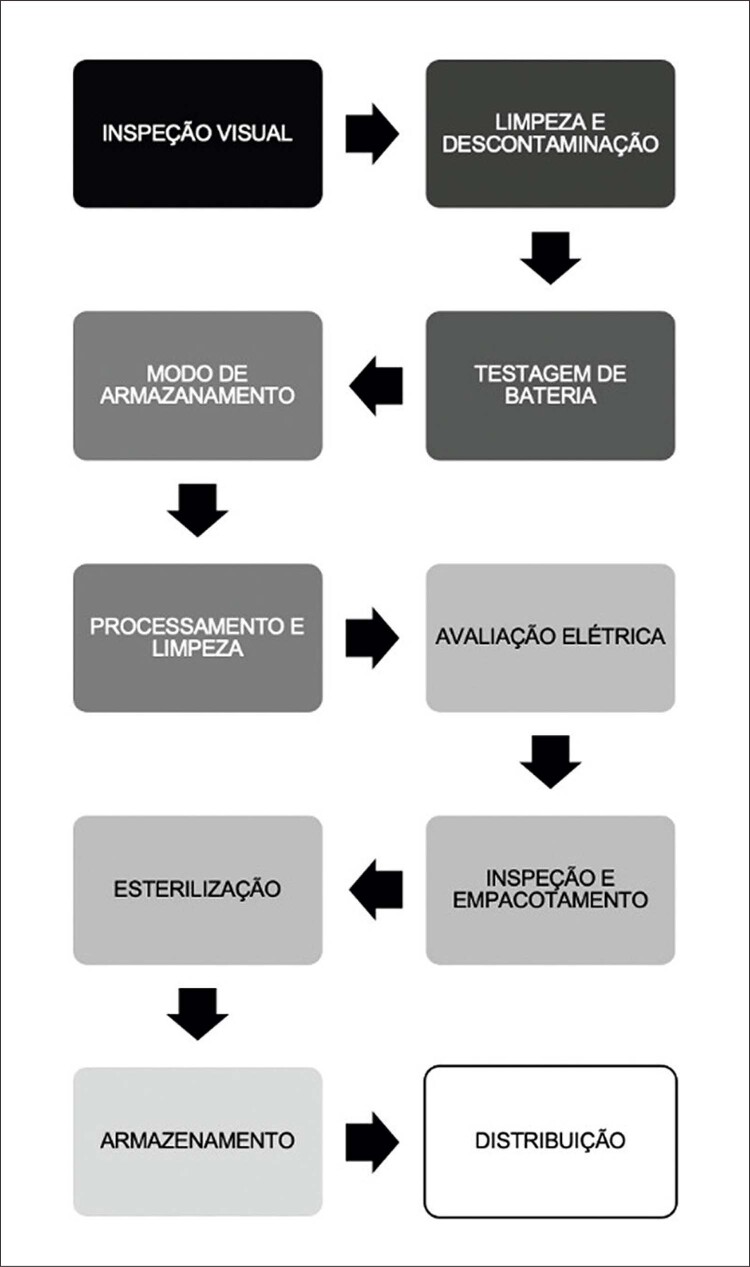
– Etapas reprocessamento.

Após serem doados por crematórios ou após extrações clinicamente indicadas, seja por infecção ou por necessidade de *upgrade,* o aparelho passa pelas seguintes fases de reprocessamento^24^ como mostrado na figura 2 :

1. Inspeção visual e, caso danificado fisicamente, é imediatamente descartado. O aparelho é interrogado rapidamente e a longevidade da bateria, testada.
2. Limpeza com spray desinfetante *Lysol* e, após isso, separação por fabricante.
3. Descontaminação:
  - O aparelho é submerso em deionização/osmose reversa (D-OR) e solução de detergente Enzol por 10 minutos ( *Advanced Sterilization Products, Irvine, Califórnia* ).
  - O aparelho é lavado com água deionizada e, posteriormente, secado.
4. As saídas dos geradores são então conectadas a um teste de 500-Ohms. Os aparelhos são então programados com uma saída de 2.5V com 1ms de largura de pulso, a uma frequência de 60bpm. Com essa programação se interroga a bateria. Se bateria é inferior a 4 anos, o aparelho é descartado. Se superior a 4 anos ou 75% da bateria, o gerador é separado de acordo com o fabricante.
5. O gerador é colocado em modo de armazenamento. Ele é desligado, se houver essa função, ou colocado em VVI com saída de 1V a 0.1ms, frequência de 30, e todos os sensores são desligados.
6. Processamento e limpeza:
  - As tampas dos parafusos são retiradas. Os parafusos são retirados, lavados com água destilada, secados e separados por fabricante.
  - Colocam-se os parafusos e o gerador em uma pia com D-OR e imersos em solução enzimática de Enzol por 3 minutos.
  - Os aparelhos são então esfregados quatro vezes com uma toalha sem fiapos. Todas as fendas são escovadas com uma escova de náilon. Conectores são escovados com limpadores de tubos enquanto imersos em água. O aparelho é inspecionado com uma lupa de 5 aumentos e, caso sejam encontrados detritos, o aparelho é escovado novamente. Se forem encontrados detritos após dois ciclos de escovação e limpeza, o aparelho é descartado.
  - Os aparelhos são colocados em D-OR. Depois de 3 minutos a pia é drenada. O processo é repetido por três vezes.
  - Lava-se novamente com água deionizada e depois seca-se com ar a 35 - 50 °C por 7 horas.
  - Se houver qualquer aparente resíduo, o aparelho é descartado.
7. Avaliação elétrica:
  - Recolocam-se novos parafusos e tampas de silicone e o aparelho é submerso em silicone medicinal quatro vezes.
8. O aparelho é reinterrogado e programado em modo de armazenamento.
9. Inspeção e empacotamento
10. Esterilização
  - Esterilização usando 100% óxido de etileno em câmara a vácuo por 8 horas.
  - Câmara de aeração por 48 horas.
11. O aparelho é então colocado em área estéril até ser enviado para reuso.

### Ética

Apesar de aparentemente seguro do ponto de vista infeccioso, como descrito anteriormente, algumas preocupações devem ser levadas em consideração: o maior risco de disfunção do dispositivo e a menor durabilidade da bateria.

Essa aparente diferença entre os aparelhos esterilizados e novos levanta uma questão: é apropriado reutilizar aparelhos não sendo esses iguais aos novos? Marca-passos podem ser exportados de países de alta renda para países de baixa e média renda mesmo sem seu uso ser permitido nos países doadores?^25^

Em bioética, justiça é dar a cada um o que é seu por direito, de forma equitativa e apropriada a uma pessoa. Uma injustiça acontece quando se nega a uma pessoa um bem a que tem direito, ou se distribui de maneira desigual.^26^ Então, não é justo oferecer um marca-passo recondicionado se há a possibilidade de adquirir um aparelho novo, haja vista a maior longevidade de bateria e o menor risco de disfunção do aparelho novo. Entretanto, caso esses aparelhos sejam oferecidos a países que não tenham uma política pública que garanta amplo acesso a eles, seria mesmo aceitável os privar desse tratamento, sabendo que, no fim das contas, a ausência de terapia para bradicardia pode ocasionar importante morbimortalidade?^27^

Mesmo em países onde haja déficits na aquisição desses aparelhos, a decisão final sobre a utilização de marca-passos reutilizados deve ser do paciente. Esse deve preencher um consentimento livre e esclarecido antes do procedimento. É ainda direito do indivíduo se negar a utilizar o aparelho recondicionado caso assim queira.^28^

Em caráter de pesquisa, é ainda necessário que haja a autorização de comitês de ética locais e que haja um plano para a continuidade do fornecimento dos produtos e acompanhamento dos pacientes caso a pesquisa se encerre.^29^

### Os fabricantes e os dispositivos reprocessados

As empresas de dispositivos sempre funcionaram em um paradigma de dispositivos de uso único. A reutilização de dispositivos não é sancionada pelos fabricantes por vários motivos. Os dispositivos reprocessados não atendem às especificações originais e por isso mesmo não podem ser garantidos pelos fabricantes originais. Há uma série de considerações legais sobre a reutilização de dispositivos de uso único que não tem precedência. A reutilização de dispositivos pode expor os fabricantes de dispositivos a possíveis litígios por parte de uma parte lesada. A indústria é altamente regulamentada e todos os seus produtos são aprovados especificamente em cada jurisdição com especificações claras. É improvável que a aplicação mais ampla da reutilização de marca-passos promova os interesses comerciais dos fabricantes.

Países de língua portuguesa e a reutilização de marca-passos

Apesar de existirem vários países de língua portuguesa e de baixa e média renda que poderiam se beneficiar, essas parcerias ainda não foram até o momento concretizadas.

Pelo nosso conhecimento não há nenhuma larga organização em países de língua portuguesa com o objetivo de reusar marca-passos para pacientes que não têm como adquirir um aparelho novo.

Situação da estimulação cardíaca em alguns países de língua portuguesa ( Tabela 2 )

**Tabela 2:** – Países de língua portuguesa e disponibilidade de dispositivos. Em vermelho, países onde dispositivos cardíacos não estão disponíveis; em amarelo os parcialmente disponíveis e em verde os amplamente disponíveis

População do País/IDH*/ GNI -PP	Disponibilidade de Dispositivos Cardíacos Eletrônicos Implantáveis (DCEI)
**Portugal** 10,3M/0,86/33 967USD	Amplamente disponível através do sistema público nacional de saúde.
**Brasil** 210M/0,765/14 263USD	Embora os atrasos no acesso ao atendimento cardíaco especializado possam acontecer, os DCEIs estão amplamente disponíveis através do sistema público nacional de saúde e de seguros privados.
**Angola** 32,87M/0,581/6 104USD	Os CDEIs não estão amplamente disponíveis. Os procedimentos são realizados nos hospitais público, militar e privado. Atrasos no sistema público de saúde são comuns.
**Moçambique** 31,26M/0,4/1 250USD	Os DCEIs não estão amplamente disponíveis. Se o paciente tem a capacidade de pagar o dispositivo, existem hospitais com capacidades cirúrgicas. Caso não, os pacientes contam com o sistema público de saúde que atualmente só tem capacidade para atender 20% da demanda.
**Cabo Verde 0,55M/0,665/7 019USD**	Os DCEIs não estão amplamente disponíveis. Se o paciente tem a capacidade de pagar o dispositivo, existem dois hospitais com capacidade cirúrgica. Caso não, os pacientes são transferidos para Portugal. Atrasos são comuns.
**São Tomé e Príncipe** 0,21M/0,62/3 952USD	Os DCEIs não estão disponíveis no momento. Não há radioscopia no país. Os pacientes são transferidos para Portugal. Atrasos são comuns.
**Guiné-Bissau** 1,98M/0,48/1 996USD	Os DCEIs não estão disponíveis no momento. Não há radioscopia no país. Os pacientes são transferidos para Portugal. Atrasos são comuns.
**Timor-Leste** 1,31M/0,606/4 440USD	Os DCEIs não estão disponíveis no momento. Não há radioscopia no país. Os pacientes são transferidos para Indonésia, Cingapura ou Malásia.

Fonte: Human Development Report Office 2020 – Nations. *HDI: Índice de desenvolvimento humano. ** GNI-PP: Renda nacional bruta per capita – *https://hdr.undp.org/en/content/latest-human-development-index-ranking*

#### Brasil

O Brasil apresenta taxas de implantes de marca-passos bem inferiores às dos países considerados desenvolvidos, bem como em relação a outros países latino-americanos.^30 , 31^ Diversos fatores podem justificar essas diferenças, como as dificuldades de acesso ao sistema de saúde de alta complexidade, o baixo número de centros de implantes em algumas regiões do Brasil e a estratificação inadequada dos pacientes que necessitam de implantes de dispositivos cardíacos eletrônicos implantáveis (DCEI).

Estima-se que o serviço público tenha mais de 234 centros cardiovasculares especializados, apesar de ser bem mais elevado o número de centros em hospitais privados. A falta de próteses para os implantes não tem se mostrado um fator presente nesses centros especializados e quando ocorre, deve-se principalmente a razões burocráticas, relacionadas à demora nas realizações das licitações públicas para as compras desses dispositivos.

No Brasil, um país de grandes dimensões e heterogeneidade no acesso aos sistemas de saúde, existem regiões em que 80% do atendimento médico é fornecido pelo setor público de saúde, enquanto em regiões mais desenvolvidas esse número fica em torno de 50 - 60%. Os pacientes, tanto do setor público como do privado, têm assegurado por lei o direito constitucional ao acesso para procedimentos de alta complexidade e ao implante de próteses cardíacas.

A Legislação Brasileira proíbe a reutilização e o reprocessamento de DCEI, como marca-passos, desfibriladores e ressincronizadores. A ANVISA, órgão regulador brasileiro, equivalente ao FDA nos Estados Unidos, delimita as normas técnicas em relação ao assunto.^32 , 33^ A ANVISA regulamentou e esclareceu que alguns materiais utilizados em eletrofisiologia e hemodinâmica pudessem ser reprocessados, desde que seguissem as instruções normativas publicadas e que o reprocessamento fosse permitido pelos fabricantes, que deveriam colocar no rótulo, “proibido reprocessar”, quando apropriado.^34^

Diversas incongruências foram encontradas nos rótulos conforme descrito por Kuniyoshi, representando a SOBRAC (Sociedade Brasileira de Arritmias Cardíacas).^35^Portanto, materiais que não tivessem no rótulo a proibição de reprocessamento poderiam ser analisados, desde que uma empresa especializada envolvida no processo atestasse a manutenção da qualidade do material reprocessado. Isso determinou grande alívio para diversos serviços de cardiologia no país, principalmente no nível público, que usualmente já reprocessavam alguns materiais e haviam sido proibidos, determinando a inviabilidade de funcionamento, considerando os baixos preços pagos principalmente pelo serviço público e elevado valor dos materiais importados.^35^

O reuso de dispositivos no Brasil foi uma prática aceitável na década de 80, quando médicos realizavam tais procedimentos em pacientes de baixa renda sem assistência à saúde garantida, porém isso nunca foi a prática padrão.

É indiscutível que um processo que leve à redução de custos e maior acesso da população a tratamentos necessários e de alta complexidade seja bem-vindo, entretanto, no contexto brasileiro, a falta de acesso aos centros especializados parece ser o maior problema que limita a ampliação desse tratamento e não a falta de aparelhos.

#### Angola

Angola conta com quatro centros de implantes de marca-passos, sendo dois privados e dois públicos, todos situados na capital do país. Possui três médicos treinados em implantes de dispositivos cardíacos, dois cardiologistas e um cirurgião.

São implantados marca-passos convencionais, porém já houve implantes de ressincronizadores com apoio de cardiologistas estrangeiros.

Em média, são implantados cerca de 30 a 35 dispositivos em cada centro privado e em torno de 100 no hospital público. Nos hospitais públicos, os procedimentos são gratuitos, com a particularidade de que, no Hospital Militar, apenas são atendidos militares e seus familiares diretos.

São várias as dificuldades enfrentadas nessa atividade, desde a falta de recursos materiais nos hospitais públicos até o custo elevado nas instituições privadas, onde apenas pessoas com convênios de empresas e algumas seguradoras (nem todas as seguradoras custeiam os procedimentos) têm acesso a cirurgia, o que faz com que a lista de espera no hospital público seja ainda maior. Acresce-se a essas dificuldades a escassez de recursos humanos.

#### Moçambique

Existem somente 25 cardiologistas, 80% dos quais localizados na capital. Só existem cardiologistas em 4 das 10 capitais provinciais, havendo, portanto, imensas áreas sem qualquer cobertura da especialidade.

Os únicos centros aptos a colocar marca-passos, um privado e outro público, localizam-se ambos na capital. Em média são colocados por ano cerca de 20 a 30 marca-passos, a maior parte deles no hospital público a doentes indigentes e a custo zero. Os marca-passos adquiridos pelo estado para serem colocados no hospital público não cobrem senão 20% das necessidades atuais e estas poderão aumentar largamente com uma campanha de detecção de casos elegíveis no nível dos centros de saúde primários, o que nunca foi feito para não criar uma expectativa que não se pode resolver.

Uma experiência anterior de uso de marca-passos recondicionados vindos da Espanha em pequena quantidade decorreu sem qualquer problema quer de aceitação ou de complicações.

#### Cabo Verde

Cabo Verde possui dois hospitais centrais e 4 regionais. Os hospitais centrais são os que oferecem cuidados mais especializados. Os doentes cujas necessidades não podem ser satisfeitas internamente, baseados num protocolo intergovernamental, são transferidos para Portugal. Porém, esse protocolo contempla uma quota anual que coloca certo limite às demandas. A primeira causa de morte é o acidente vascular cerebral e a primeira causa de incapacidade é a doença cardíaca isquêmica, sugerindo que a melhora da saúde cardiovascular poderia ter importante impacto populacional.

Desde 2016, baseado num projeto para implantes de marca-passos, médicos locais especializados têm realizado implantes de forma esporádica, sempre com os próprios doentes assumindo o custo do DCEI. Tem-se um registro de quase 80 doentes aguardando cirurgias e estima-se que em média 50 doentes por ano venham a precisar de marca-passos. Muitos dos que estão a espera nunca são chamados para implantes em Portugal, outros nem entram na lista. Há uma necessidade de aquisição de DCEI, pois o país já apresenta capacidade técnica para tal.

#### São Tomé e Príncipe

O único serviço de cardiologia do país está localizado no Hospital Ayres de Menezes, fundado em 2012, e a infraestrutura montada oferece exames não invasivos como ecocardiograma transtorácico, teste ergométrico e Holter. Existe apenas um cardiologista em todo o país.

Entre 2016 e 2021, foram diagnosticados, em média, dez bloqueios atrioventriculares com implante de marca-passo por ano. Por não haver estrutura local para implantação desses dispositivos, esses pacientes são transferidos para Portugal. No entanto, a transferência para Portugal é complexa e raramente pode ser feita com urgência. O tempo médio de espera para a saída desses pacientes é de aproximadamente 6 meses; portanto, muitos deles acabam morrendo antes de terem a cirurgia.

#### Guiné Bissau

O único serviço de cardiologia é o Hospital Nacional. Atualmente, não há infraestrutura ou pessoal treinado para realizar as cirurgias do DCEI. Os doentes são transferidos para Portugal, mas o tempo de espera pode chegar até dois anos.

#### Timor-Leste

Existem quatro hospitais no país com serviços de cardiologia, porém nenhuma intervenção cardíaca é realizada no país. Os pacientes com necessidade de DCEI são transferidos para a Indonésia, Malásia ou Cingapura, os custos são cobertos pelo governo e os pacientes são transferidos em uma semana.

## Conclusões

Há uma enorme disparidade entre países de baixa e média renda e os países de alta renda, especialmente quando se envolve a estimulação cardíaca artificial. O reuso de marca-passo tem se mostrado seguro, ético e eficaz para aqueles que necessitam de dispositivos cardíacos implantáveis e não têm como adquiri-los. Parte dos países de língua portuguesa, especialmente na África, necessitam de uma resposta imediata que beneficie os seus inúmeros pacientes que padecem de arritmias tratáveis. Entretanto, cada nação deve ter uma ampla discussão legal e ética antes de aprovar essa técnica

## References

[1] Baman TS, Eagle KA (2011). Cardiac device reutilization: Is it time to “go green” in underserved countries?. Pacing Clin Electrophysiol.

[2] Ward C, Henderson S, Metcalfe NH (2013). A short history on pacemakers. Int J Cardiol.

[3] Mond HG, Proclemer A (2011). The 11th world survey of cardiac pacing and implantable cardioverter-defibrillators: Calendar year 2009 - A world society of Arrhythmia’s project. Pacing Clin Electrophysiol.

[4] Sinha SK, Sivasambu B, Yenokyan G, Crawford TC, Chrispin J, Eagle KA (2018). Worldwide pacemaker and defibrillator reuse: Systematic review and meta-analysis of contemporary trials. Pacing Clin Electrophysiol.

[5] Runge MW, Baman TS, Davis S, Weatherwax K, Goldman E, Eagle KA (2017). Pacemaker recycling: A notion whose time has come. World J Cardiol.

[6] Mond HG, Mick W, Maniscalco BS (2009). Heartbeat International: Making “poor” hearts beat better. Hear Rhythm.

[7] Baman TS, Romero A, Kirkpatrick JN, Romero J, Lange DC, Sison EO (2009). Safety and Efficacy of Pacemaker Reuse in Underdeveloped Nations. J Am Coll Cardiol.

[8] Araujo H, Melo C (2014). Homenagem Especial – Seymour Furman. Relampa.

[9] Panja M, Sarkar C, Kumar S, Kar A, Mitra S, Sinha D (1996). Reuse of Pacemaker. Indian Heart J.

[10] European Society of Cardiology (1998). Policy document re-use of devices in cardiology. Eur Hear J.

[11] Hariprasad MK (1982). Reuse of cardiac pacemakers. N Engl J Med.

[12] Vlay SC (2018). Barriers to pacemaker and ICD recycling. Pacing Clin Electrophysiol.

[13] Selvaraj RJ, Sakthivel R, Satheesh S, Pillai AA, Sagnol P, Jouven X (2017). Reuse of pacemakers, defibrillators and cardiac resynchronisation devices. Heart Asia.

[14] Baman TS, Crawford T, Sovitch P, Meier P, Sovitch N, Gakenheimer L (2012). Feasibility of postmortem device acquisition for potential reuse in underserved nations. Hear Rhythm.

[15] Gakenheimer L, Romero J, Baman TS, Montgomery D, Smith CA, Oral H (2014). Cardiac implantable electronic device reutilization: Battery life of explanted devices at a tertiary care center. PACE - Pacing Clin Electrophysiol.

[16] Hughey AB, Baman TS, Eagle KA, Crawford TC (2013). Pacemaker reuse: an initiative to help those in underserved nations in need of life-saving device therapy. Expert Rev Med Devices.

[17] Baman TS, Kirkpatrick JN, Romero J, Gakenheimer L, Romero A, Lange DC (2010). Pacemaker reuse: An initiative to alleviate the burden of symptomatic bradyarrhythmia in impoverished nations around the world. Circulation.

[18] Kirkpatrick JN, Papini C, Baman TS, Khota K, Eagle KA, Verdino RJ (2010). Reuse of pacemakers and defibrillators in developing countries: Logistical, legal, and ethical barriers and solutions. Hear Rhythm.

[19] Sethi KK, Bhargava M, Pandit N, Mohan JC, Arora R, Khanna SK (1992). Experience with recycled cardiac pacemakers. Indian Heart J.

[20] Costa R, Moreira LF, Pêgo-Fernandes PM, Martinelli MM, Stolf NA, Verginelli G (1983). Reutilização de geradores de marca-passo. Arq Bras Cardiol.

[21] Baman TS, Meier P, Romero J, Gakenheimer L, Kirkpatrick JN, Sovitch P (2011). Safety of pacemaker reuse a meta-analysis with implications for underserved nations. Circ Arrhythmia Electrophysiol.

[22] Enache B, Șoșdean R, Macarie R, Dodinot B, Pescariu S (2019). Assessing the safety of implantable cardioverter-defibrillator reuse—A retrospective case-control study. Pacing Clin Electrophysiol.

[23] Khairy TF, Lupien MA, Nava S, Baez FV, Ovalle FS, Ochoa NE (2020). Infections Associated with Resterilized Pacemakers and Defibrillators. N Engl J Med.

[24] Crawford TC, Allmendinger C, Snell J, Weatherwax K, Lavan B, Baman TS (2017). Cleaning and Sterilization of Used Cardiac Implantable Electronic Devices With Process Validation: The Next Hurdle in Device Recycling. JACC Clin Electrophysiol.

[25] Aragam KG, Baman TS, Kirkpatrick JN, Goldman EB, Brown AC, Crawford T (2011). The ethics of pacemaker reuse: might the best be the enemy of the good?. Heart.

[26] Siurana JC (2010). Los principios de la bioética y el surgimiento de una bioética intercultural. Veritas.

[27] Hutchison K, Sparrow R (2018). Ethics and the cardiac pacemaker: More than just end-of-life issues. Europace.

[28] Artal R, Rubenfeld S (2017). Ethical issues in research. Best Pract Res Clin Obstet Gynaecol.

[29] Laman M, Pomat W, Siba P, Betuela I (2013). Ethical challenges in integrating patient-care with clinical research in a resource-limited setting: Perspectives from Papua New Guinea. BMC Med Ethics.

[30] Galvao S, Vasconcelos J, Pachon-Mateos J (2004). Registro Brasileiro de Marcapassos (RBM) no Ano de 2003. Dez Anos de RBM. Reblampa.

[31] Pachón-mateos JC, Pereira WL, Duarte W, Junior B, Mateos CP, Indalécio E (2013). RBM - Registro Brasileiro de Marcapassos , Ressincronizadores e Desfibriladores. Relampa.

[32] Saúde. M da, Sanitária, ANVISA (2006). Resolucao - RDC No 2606, de 11 de agosto de 2006. Diário Of da União.

[33] Ministério da Saúde, Agência Nacional de Vigilância Sanitária (2006). Resolucao - RDC No 156, de 11 de agosto de 2006. Diário Of da União.

[34] ANVISA Nota Técnica n° 001/2013/GEMAT/GGTPS/ANVISA. Infor.

[35] Kuniyoshi RR, Sternick EB, Nadalin E, Hachul DT (2017). Reprocessamento de produtos médicos em eletrofisiologia. Arq Bras Cardiol.

