# Supplementary material for: Reprocessamento de Marca-Passos em Países de Língua Portuguesa: Uma Reflexão Clínica
Source: Arq Bras Cardiol. 2023 Mar 1;120(2):e20210941. [Article in Portuguese] doi: 10.36660/abc.20210941 (PMC12080717; doi:10.36660/abc.20210941)
Supplement: Translation

# Translation: Pacemaker Reuse in Portuguese Speaking Countries: A Clinical Reflection

**Authors:** Neiberg de Alcantara Lima, Eduardo Arrais Rocha, Albertino Damasceno, Ieda Prata Costa, José Ribeiro Bunda Ricardo, Fernando Jorge Lopes, Luis Dias, Miryan Bandeira dos Prazeres Cassandra Soares, Eric Puroll, Kim A. Eagle, Thomas C. Crawford

**Affiliations:** 1 EUA Wayne State University School of Medicine – Internal Medicine, Detroit, Michigan – EUA; 2 Brasil Universidade Federal do Ceará – Hospital Universitário Walter Cantídio – Programa de Pós-graduação em Ciências Cardiovasculares da Faculdade de Medicina da UFC, Fortaleza, CE – Brasil; 3 Moçambique Universidade Eduardo Mondlane – Faculdade de Medicine, Maputo – Moçambique; 4 Angola Clinica Girassol, Luanda – Angola; 5 Hospital Dr. Baptista de Sousa, Mindelo – Cabo Verde; 6 Hospital Agostinho Neto, Praia – Cabo Verde; 7 São Tomé e Príncipe Hospital Dr. Ayres de Menezes, São Tomé – São Tomé e Príncipe; 8 EUA University of Michigan Medicine, Ann Arbor, Michigan – EUA; 9 EUA University of Michigan Medicine – Frankel Cardiovascular Center, Ann Arbor, Michigan – EUA

**Keywords:** Cardiac Pacing Artificial/trends, Pacemaker, Artificial, Defibrillators, Implantable, Public Health,Administration, Health Status Disparities

## Abstract

There is a gap between high-income countries and others in terms of access to medical cardiac devices, such as pacemakers and implantable cardioverter defibrillators. Costs are one of the main barriers to the use of cardiac devices in these countries. There are international initiatives that aim to reduce the gap. The reuse of pacemakers has been discussed as a possible alternative to this problem. The concept of reusing pacemakers is not new; however, recent studies have proven to be safe, ethical, and effective for those who need cardiac implantable electronic devices and cannot afford them. Part of the Portuguese-speaking countries, especially in Africa, need an immediate response that benefits their countless patients who suffer from treatable arrhythmias.

## Introduction

Artificial cardiac pacing and electrophysiology are well established and internationally recognized medical activities, but there is still a gap between high-income countries and others in terms of access to medical devices. The reuse of pacemakers has been discussed as a possible alternative to this problem.^1^However, even though there is an evident need in some areas, low- and middle-income Portuguese-speaking countries still do not have defined public policies on this subject or started broad discussions on this topic in their medical societies. This review proposes to discuss what is known about this theme, promoting the possibility of discussing the implementation of pacemaker reuse, particularly in regions with greater difficulty in acquiring these cardiac devices.

### Disparities in artificial cardiac pacing

Since the 1950s, when the first pacemakers were implanted, there has been an unprecedented advance in the treatment of heart disease.^2^Although the technology has greatly advanced, it is still expensive and inaccessible for many low- and middle-income countries. For example, the annual pacemaker implant rate is more than 700 per million inhabitants in France, Sweden, and the United States, while it is less than 7 per million in Pakistan, the Philippines, and Indonesia, and less than 3 per million in Africa. Based on those numbers, it is estimated that up to one million people die each year without access to therapy for severe bradyarrhythmia in the world.^3 - 5^Cost is one of the main barriers to the use of cardiac devices in these countries. A pacemaker costs up to US$2,500.00, which is more than the per capita income of many low- and middle-income countries.^5^The lack of public health policies for non-infectious diseases, professionals trained to recognize and treat cardiac arrhythmias, as well as access to specialized hospitals and surgical infrastructures are other possible reasons.

There are initiatives that aim to reduce the gap between high-income and low- and middle-income countries, such as Heartbeat International (https://heartbeatsaveslives.org/), an organization that distributes pacemakers close the manufacturer expiration date, which are generously donated by the device industry.^6^

The *Project My Heart Your Heart* (PMHYH) is a collaboration between US citizens, physicians, and funeral directors, University of Michigan, NEScientific, and World Medical Relief (WMR). WMR is a Detroit-based, non-profit philanthropic organization whose mission is to improve the well-being of the indigent in low- and middle-income countries through the distribution of donated medical supplies, equipment, and medications.^7^The purpose of this collaboration is to determine whether pacemaker reuse is safe and scalable.

Prior to the approval of international clinical trials, the PMHYH spent the first years providing the framework for this idea to be supported by all those it would impact.

In more than 10 years, the PMHYH has already benefited countless patients who received, free of charge, reconditioned pacemakers sent to countries such as the Nigeria, the Philippines, Venezuela, Kenya, and Sierra Leone, among others. The program also provides training and follow-up for local professionals and patients. For more information, visit http://www.myheartyourheart.org.

### Reuse of cardiac devices

The concept of reusing pacemakers is not new.^1^Even though it was never the standard of care, they have already been reused in Brazil and India in the 1980s and 1990s, respectively.^8 , 9^In the 1990s, Sweden reused around 5% of their pacemakers.^10^Attempts to donate pacemakers taken from the deceased donors to the developing countries by American physicians date back to the 1980s.^11^Reused implantable cardioverter defibrillators (ICDs) and biventricular pacemakers have also been implanted in Africa, but in a smaller scale.^12 , 13^

Potential sources of pacemaker donation are: post-mortem and post-extraction. One study showed that more than three thousand devices are removed by American crematoriums, and at least a fifth of those had a battery longer than 4 years. A new device has a battery life of up to 12-14 years, which varies by programming and the device model.^14^More than 60,000 devices are explanted per year in the United States, and it is estimated that around 20% of these could be reused, creating a total of 10 to 12 thousand devices per year.^15 - 17^

Although donating pacemaker generators is feasible, reusing leads is not feasible. Leads are, in general, difficult to be removed due to the fibrosis process caused over time after their implantation, and their extraction is potentially harmful to them. Even if properly removed, the lead sterilization process is also challenging.^18^However, pacemaker leads are much less expensive than pulse generators, and can usually be purchased by patients in low- and middle-income countries.

### Safety

In relation to the use of a reprocessed device, the main concerns are the risk of infection and the risk of device dysfunction ( Table 1 ).

**Table 1:** – Safety of reprocessed pacemaker

Study ID	Years of Study	Type of study	Country	Total of patients with reused CIED	Infection rate (%)	CIED Mafunction rate (%)	Mortality
Panja M et al. Indian Heart J, 1996^*9*^	1976-1992	Observational	India	642	11.8% *Mostly when CIED were reused in the same patient after being sterilized.*	3.1% - H *igh threshold*	Not reported
Baman T et al. Circ Arrhythm Eletrophysiol 2011^*21*^	2011	Metanalysis	India, Romania, Sweden, Brazil, Hungary, Israel, Australia, Finland, Norway, Canada, Holland, the Philippines, Italy	2270	*1.76%. Not different than the control group.*	*0.5%. Higher than new devices*	None
Enache et al. PACE, 2019^*22*^	2001-2012	Retrospective. Case control.	Romania	157	*5% Not different than the control group*	None	None
Sethi et al. Indian Heart J. 1992^*19*^	1979-1991	Observational	India	42	2.3%.	None	None
Khairy et al. NEJM, 2020^*23*^	2003-2017	Controlled, prospective study.	Cuba, Dominican Republic, Ecuador, Guatemala, Honduras, and Mexico	1051	*2% Not different than the control group*	None	None
Baman et al. JACC 2009^*7*^	2008	Case series	Philippines	12	None	None	None
Sinha et al. PACE 2018^*4*^	2009-2017	Metanalysis	Philippines, Nicaragua, India, Mexico, China, South Africa, Romania, India.	856	*2.1%. Not different than the control group*	*0.2% Not different than the control group*	None

Regarding infection, there is vast literature showing that reconditioned devices are safely reused. According to one report, between 1979 and 1991, forty-two devices were donated and reused in India after being explanted, mostly due to generator infection, and after a 3-year follow-up, only 2 patients had infectious complications.^19^

In the 1980s, in the city of Sao Paulo, 22 devices were reused after chemical decontamination and the use of ethylene oxide. Of those, only one patient had complications due to electromagnetic inhibition of the generator, which happened 2 months after implantation.^20^

In 2011, a meta-analysis on the safety of device reuse, with pacemaker infection as the primary endpoint, included 18 papers published from 1988 to 2008, conducted with a total of 2,270 patients. Device reprocessing methods were not homogeneous. The infection rate was only 1.97%. Of these 18 studies, 5 were controlled with a total of 913 patients included, and showed no difference in infection compared to 6,697 new device implantations.^21^

A nonrandomized study of reused ICDs compared 157 patients who received reused devices and 114 patients who received new devices. There was no statistical difference between the groups regarding infections, device malfunction, or unexpected battery depletion.^22^

In 2020, a group from the University of Montreal, together with international collaboration, published an important study in the New England Journal of Medicine which compared 1,051 pacemakers and ICD devices reused in Mexico, the Dominican Republic, Guatemala, and Honduras, with 3,131 new pacemaker implantations performed in Canada. Baseline characteristics of control patients in Canada were adjusted for the study group. The sterilization protocol in that study was not described. No statistical difference was observed in device-related mortality or infection between the groups.^23^

The same 2011 meta-analysis showed a secondary outcome of the assessment of device malfunction and included seventeen papers with a total of 2,150 reused devices, revealing only 13 device malfunctions, described as “technical errors” (n=5), premature battery depletion (n =1), spontaneous reprogramming (n=1), electromagnetic inhibition (n=1), pectoral muscle inhibition (n=1), screw abnormalities (n=3), and spontaneous ventricular tachycardia (n=1). In four controlled studies of 793 reused pacemakers and 2,200 new implants, there was an increased risk of malfunction in the reused devices.^21^

The major disadvantage of a reused device, quite naturally, is a shorter life span when compared with a new device, but this is expected since they have already been used.^21^The PMHYH only reprocesses pacemakers if the battery longevity exceeds four years or has 75% of the total manufactored battery.

### Reprocessing technique

There are validated techniques for reprocessing pacemakers, aiming to minimize the risk of infection and dysfunction of the devices to be reimplanted. Here, we describe a validated technique developed by our group at University of Michigan and published in the Journal of American College of Cardiology in 2017 ( Figure 1 ).^24^

**Figure 1:** – My Heart Your Heart lab in Detroit Project.

After being donated by crematoriums or after clinically indicated extractions in hospitals, either due to infection or due to the need for an upgrade, the device passes through the following phases of reprocessing,^24^as shown in Figure 2 :

**Figure 2:** – Device Reprocessing steps.

1. Visual inspection, if damaged the device is discarded. The device is inspected, and the longevity of the battery is tested.
2. Cleaning with Lysol disinfectant spray and sorting according to the manufacturer.
3. Decontamination:
  - The device is immersed in deionization/reverse osmosis (RO-DI) and Enzol detergent solution for 10 minutes (Advanced Sterilization Products, Irvine, California).
  - The device is washed with deionized water and then wiped dry.
4.  The generator outputs are then connected to a 500-Ohm test load. The devices are programmed with a 2.5V output with a 1ms pulse width, at a rate of 60. With this programming, the battery is inspected. If the remainder of the battery is less than 4 years, the device is discarded. If longer than 4 years of battery or 75% of the battery, the generator is separated according to the manufacturer.
5. The generator is configured to storage settings. It is turned off if this function is activated or placed in VVI with 1V output at 0.1ms, with a frequency of 30 and all sensors turned off.
6. Processing and cleaning:
  - The screw covers are removed. The screws are removed, washed with distilled water, wiped dry, and sorted by manufacturer.
  - The screws and generator are placed in a sink with RO-DI and immersed in Enzol’s enzymatic solution for 3 minutes.
  - The devices are then rubbed four times with a lint-free towel. All devices are brushed with a nylon brush. Connectors are brushed with tube cleaners while immersed in water. The appliance is inspected with a 5x magnifying glass; if debris are found, the appliance is brushed again. If debris are found after two cycles of brushing and cleaning, the device is discarded.
  - Devices are placed in RO-DI again. After 3 minutes, the sink is drained. The process is repeated three times.
  - It is washed again with deionized water and then air dried at 35-50°C for 7 hours.
  - If there is any apparent residue remaining, the device is discarded.
7. Electrical testing:
  - The screws are replaced, and a new silicone cover is placed; the device is then dipped four times in medical grade silicone.
8. The device is re-inspected and programmed for storage in a non-pacing mode, if available, as described above.
9. Inspection and packaging.
10. Sterilization
  - Sterilization using 100% ethylene oxide in a vacuum chamber for 8 hours.
  - Aeration chamber for 48 hours.
11. The device is then placed in a sterile area to wait for shipping.

### Ethics

Although apparently safe from the infectious standpoint, as described above, some concerns should be taken into consideration: the increased risk of device malfunction and a shorter battery life.

This apparent difference between reprocessed and new devices raises a question: Is it appropriate to reuse devices if they are inferior to new devices? Can pacemakers be exported from high income countries to low- and middle- income countries if they are not allowed for patient use in the donor countries?^25^

In bioethics, justice is giving everyone what is rightfully theirs, equitably and appropriately for a person. Injustice occurs when a person is denied a good that he/she is entitled to or when it is distributed unequally.^26^Therefore, it is not fair to offer a reprocessed pacemaker if there is a possibility of providing a new device, given the longer battery life and the lower risk of malfunction of the new device. However, if these devices are offered to countries that do not have a public policy that guarantees reasonable access to them, would it really be acceptable to deprive them of this treatment, knowing that the absence of therapy for bradycardia can cause significant morbidity and mortality?^27^

Even in countries where the provision of new devices is severely limited, the final decision on the use of reused pacemakers must be made by the patient. The patient must complete an informed consent prior to the procedure. The patient has the right to refuse the refurbished device.^28^

In terms of research, it is still necessary to have the authorization of the local IRBs and a plan for the continuity of the supply of products and monitoring of patients when the research ends.^29^

### Manufacturers and reprocessed pacemakers

Device companies have always functioned in a paradigm of single use devices. Device reuse is not sanctioned by the manufacturers for a multitude of reasons. Reprocessed devices do not meet the original specifications and *ipso facto* cannot be guaranteed to be performed by the original manufacturers. There is a host of legal considerations concerning the reuse of single use devices, which is without precedence. Reusing devices could expose device manufacturers to possible litigation by an injured party. The industry is highly regulated, and all their products are specifically approved in each jurisdiction with clear specifications. It is unlikely that broader application of pacemaker reuse would advance commercial interests of the manufacturers.

### Portuguese-speaking countries and the reuse of pacemakers

Although there are several Portuguese-speaking low- and middle-income countries that could be benefited, partnerships with projects that provide these devices have not yet been implemented.

To the best of our knowledge, there are no large organizations in Portuguese-speaking countries whose goal is pacemaker reuse to benefit patients who cannot afford a new device.

Cardiac Stimulation in Portuguese-speaking countries ( Table 2 ).

**Table 2:** – Portuguese speaking country and device availability. Countries where devices are not available in red, partially available in yellow, and widely available in green

Country Population/HDI*/GNI -PP	CIED Availability
**Portugal** 10.3M/0.86/33 967USD	Widely available through the national public health system.
**Brazil** 210M/0.765/14 263USD	Even though delays in the access to specialized cardiac care may happen, CIEDs are widely available through the national public health system and private insurances.
**Angola** 32.87M/0.581/6 104USD	CIEDs are not widely available. Procedures are performed in the public, military, and private hospitals. Delays in the public health system are common.
**Mozambique** 31.26M/0.4/1 250USD	CIEDs are not widely available. If the patient has the ability to pay the device, there are hospitals with surgery capabilities. If not, patients rely on the public health system that currently only has the capability to attend to 20% of the demand.
**Cape Verde 0.55M/0.665/7 019USD**	CIEDs are not widely available. If the patient has the ability to pay the device, there are two hospitals with surgery capabilities. If not, patients are transferred to Portugal.
**Saint Thomas and Prince** 0.21M/0.62/3 952USD	CIEDs are not currently available. No fluoroscopy machine in the country. Patients are transferred to Portugal. Delays are common.
**Guinea-Bissau** 1.98M/0.48/1 996USD	CIEDs are not currently available. No fluoroscopy machine in the country. Patients are transferred to Portugal. Delays are common.
**East Timor** 1.31M/0.606/4 440USD	CIEDs are not currently available. No fluoroscopy machine in the country. Patients are transferred to Indonesia, Singapore, or Malaysia.

Source: Human Development Report Office 2020 - United Nations. *HD: Human development index. ** GNI-PP: Gross national income per capita – *https://hdr.undp.org/en/content/latest-human-development-index-ranking*

#### Brazil

Brazil presents rates of pacemaker implants that are much lower than those considered to be high-income, as well as in relation to Latin American countries.^30 , 31^Several factors can justify these differences, such as the difficulties in accessing more specialized facilities, the low number of pacemaker implant centers in some regions of Brazil, and the inadequate stratification of patients who need cardiac implantable electronic devices (CIED).

It is estimated that the public health system has more than 234 specialized cardiovascular centers; however, there are plenty of private hospitals. The lack of CIED has not proven to be a factor present in these specialized centers, and when it does occur, it is mainly due to bureaucratic reasons, related to the delay in carrying out public tenders for the purchase of these devices.

In Brazil, a geographically large country with a broad heterogeneity in access to health care, there are regions where 80% of the medical care is provided by the public health system, while in more developed regions of the country, this number is around 50- 60%. Patients, both from the public and private sectors, have the constitutional right to have access to highly complex procedures and to the implantation of cardiac prostheses guaranteed by law.

Brazilian legislation prohibits the reuse and reprocessing of CIED, such as pacemakers, defibrillators, and CRT devices. ANVISA, a Brazilian regulatory body, equivalent to the FDA in the United States, defines the technical standards in relation to the subject.^32 , 33^ANVISA regulated and clarified that some materials used in electrophysiology and the cath lab could be reprocessed, as long as they followed the published normative instructions and were allowed by the manufacturers, who should put on the label, “no reprocessing”, when appropriate.^34^

Several inconsistencies in the labels were found as described by Kuniyoshi, in a work carried out representing SOBRAC (Brazilian Society of Cardiac Arrhythmias).^35^Therefore, materials that did not have a reprocessing ban on the label could be analyzed, as long as a specialized company involved in the process attests to the maintenance of the quality of the reprocessed material. This brought great relief to several cardiology services in the country, mainly at the public level, which usually had already reprocessed some materials and had been banned, making them unfeasible, considering the low prices paid mainly for the public service and the high value of imported materials.^35^

The reuse of pacemakers in Brazil was an accepted practice in the 1980s, especially by physicians attending to low-income patients whose medical insurance did not cover such procedures; however, it was never the standard of care.

It is indisputable that a process that leads to cost reduction and greater access to the population of necessary and highly complex treatments are welcome, but in the Brazilian context, the lack of access to specialized implantation centers seems to be the biggest problem that limits the expansion of this treatment and not the lack of CIEDs.

#### Angola

Angola has only four pacemaker implantation centers, two private and two public, all located in the country’s capital. It has three doctors trained in the implantation of cardiac devices, two cardiologists, and a surgeon.

Pacemakers are implanted in these hospitals; however, only a few CRT devices have been implanted, and this occurred thanks to the support of foreign cardiologists. On average, 30 to 35 devices are implanted in each private center and around 100 in the public hospital a year. In public hospitals, the procedures are paid by the government, but in the Military Hospital, only military staff and their immediate relatives can receive medical care.

There are several difficulties in this activity in Angola, from the lack of material resources in public hospitals to the high cost in private institutions where only people with business agreements and some insurance policies have access to these procedures; this increases the waiting list in public hospitals. There is also a scarcity of trained professionals.

#### Mozambique

There are only 25 cardiologists, 80% of those are located in the capital. There are only cardiologists in 4 of the 10 provincial capitals, and there is, therefore, a large area with no coverage of the specialty.

The only centers able to place pacemakers are both located in the capital, one is private and the other is public. On average, around 20 to 30 pacemakers a year are placed, most of them in the public hospital for the indigent patients and at no cost. Pacemakers paid by the state only attend to 20% of the current needs, and these could greatly increase with a campaign to detect eligible cases in primary health centers, which was never done to not create an expectation that cannot be consistently met.

The country had a previous experience of using reconditioned pacemakers donated from Spain and had no major issues in acceptance or complications.

#### Cape Verde

Cape Verde has two central and 4 regional hospitals. Central hospitals are those that offer specialized care. Patients whose needs cannot be met nationally, based on an intergovernmental protocol, are transferred to Portugal. However, this protocol includes an annual quota that places a certain limit on demands. The most common cause of death is stroke, and the most common cause of disability is ischemic heart disease, suggesting that improvement of the cardiovascular care here could have a significant impact on the population.

Since 2016, based on a project for pacemaker implants, local cardiologists have performed CIED implants sporadically, only if the patients assume the cost of the CIED. There is a list of nearly 80 patients awaiting surgery, and it is estimated that 50 patients per year will need pacemakers. Many of those will never be called for implants in Portugal. Thus, there is a need to acquire CIED, as the country already has trained physicians capable of performing these procedures.

#### St Thomas and Prince

The only cardiology service in the country is located at Hospital Ayres de Menezes, which was founded in 2012; the infrastructure set up offers non-invasive tests, such as transthoracic echocardiograms, exercise testing, and Holter. There is only one cardiologist in the entire country.

Between 2016 and 2021, an average of ten atrioventricular blocks requiring pacemaker implantation per year were diagnosed. Since there is no local structure to implant these devices, these patients are transferred to Portugal. However, the transfer to Portugal is complex, and it can rarely be done urgently. The average waiting time for these patients to leave is approximately 6 months; therefore, many of them end up dying before having the chance to undergo surgery.

#### Guinea-Bissau

The only cardiology service is the National Hospital. Currently, there is no infrastructure or trained staff to perform CIED surgeries. Patients are transferred to Portugal, but the waiting time can be up to two years.

#### East-Timor

There are four hospitals in the country with cardiology services, but no cardiac intervention is performed in the country. Patients in need of CIED are transferred to Indonesia, Malaysia, or Singapore. The costs are covered by the government, and patients are transferred within a week.

## Conclusions

There is a huge gap between low- and middle-income countries and high-income countries, especially when it involves artificial cardiac stimulation. Pacemaker reprocessing has proven to be safe, ethical, and effective for those who need cardiac implantable electronic devices and cannot afford them. Part of the Portuguese-speaking countries, especially in Africa, need an immediate response that benefits their countless patients who suffer from treatable bradycardias. Each country should, however, conduct a legal and ethical discussion on this matter.
